# Evaluation of a novel real-time PCR test based on the *ssrA *gene for the identification of group B streptococci in vaginal swabs

**DOI:** 10.1186/1471-2334-9-148

**Published:** 2009-09-04

**Authors:** Martina Wernecke, Ciara Mullen, Vimla Sharma, John Morrison, Thomas Barry, Majella Maher, Terry Smith

**Affiliations:** 1 Molecular Diagnostics Research Group, National Centre for Biomedical Engineering Science, Galway, Ireland; 2 Microbiology, School of Natural Sciences, National University of Ireland, Galway, Ireland; 3 Department of Obstetrics and Gynaecology, University College Hospital Galway, Ireland

## Abstract

**Background:**

Despite the implementation of prevention guidelines, early-onset group B streptococci (GBS) disease remains a cause of neonatal morbidity and mortality worldwide. Strategies to identify women who are at risk of transmitting GBS to their infant and the administration of intrapartum antibiotics have greatly reduced the incidence of neonatal GBS disease. However, there is a requirement for a rapid diagnostic test for GBS that can be carried out in a labour ward setting especially for women whose GBS colonisation status is unknown at the time of delivery. We report the design and evaluation of a real-time PCR test (*RiboSEQ *GBS test) for the identification of GBS in vaginal swabs from pregnant women.

**Methods:**

The qualitative real-time PCR *RiboSEQ *GBS test was designed based on the bacterial *ssrA *gene and incorporates a competitive internal standard control. The analytical sensitivity of the test was established using crude lysate extracted from serial dilutions of overnight GBS culture using the IDI Lysis kit. Specificity studies were performed using DNA prepared from a panel of GBS strains, related streptococci and other species found in the genital tract environment. The *RiboSEQ *GBS test was evaluated on 159 vaginal swabs from pregnant women and compared with the GeneOhm™ StrepB Assay and culture for the identification of GBS.

**Results:**

The *RiboSEQ *GBS test is specific and has an analytical sensitivity of 1-10 cell equivalents. The *RiboSEQ *GBS test was 96.4% sensitive and 95.8% specific compared to "gold standard" culture for the identification of GBS in vaginal swabs from pregnant women. In this study, the *RiboSEQ *GBS test performed slightly better than the commercial BD GeneOhm™ StrepB Assay which gave a sensitivity of 94.6% and a specificity of 89.6% compared to culture.

**Conclusion:**

The *RiboSEQ *GBS test is a valuable method for the rapid, sensitive and specific detection of GBS in pregnant women. This study also validates the *ssrA *gene as a suitable and versatile target for nucleic acid-based diagnostic tests for bacterial pathogens.

## Background

Group B *Streptococcus *(GBS) (*Streptococcus agalactiae*) is one of the leading causes of neonatal morbidity and mortality in the developed world. Early-onset GBS disease occurs within the first week of life and is associated with neonatal sepsis, pneumonia and meningitis. The mortality rate averages at 6.5% for early-onset GBS cases and for infected preterm infants it rises to 22.7% [1].

Approximately 10 - 40% of pregnant women carry GBS asymptomatically in their vagina or rectum [2-4]. Transmission to the infant occurs vertically during labour via fetal aspiration of infected amniotic fluid or during passage through the birth canal. Since the implementation of intrapartum antibiotic prophylaxis (IAP) for the prevention of GBS, the incidence of early-onset GBS disease in neonates has decreased significantly although resistance to certain antimicrobials has been reported [5]. Studies in the US show that incidence has declined from 1.5/1000 live births in 1990 [6] to 0.32/1000 live births in 2003 [1]. In the UK, early-onset GBS disease is reported to occur in 0.5/1000 births [7].

Practices recommended by public health authorities for the identification of at-risk women who should receive antibiotic treatment during labour vary internationally. Centres for Disease Control and Prevention (CDC) guidelines for the prevention of perinatal GBS disease recommend a universal, microbiological culture-based prenatal screening strategy at 35 - 37 weeks' gestation combined with IAP for GBS-colonised women [2]. Conversely, the risk factor approach relies on the presence of one or more of the following intrapartum factors as indicators for increased risk of neonatal GBS infection: previous infant with early-onset GBS disease, premature labour, prolonged rupture of membranes and fever [7]. A comparison of the two strategies showed that the culture-based approach was almost 50% more effective in preventing early-onset GBS disease [8]. The UK-based Royal College of Obstetricians and Gynaecologists (RCOG) opposes the practice of universal screening and widespread use of intrapartum antibiotics due to a lack of clear evidence of the effectiveness of such practices in controlling the incidence of neonatal sepsis [7]. A recent UK study which looked at the cost-effectiveness of prenatal screening and treatment strategies for the prevention of GBS and other bacterial infections in early infancy proposed that treatment of all pre-term and high-risk term groups would be beneficial [9].

Perinatal GBS disease prevention practices have been successful in lowering neonatal GBS disease by 50 - 80% [10,11]. Nevertheless, early-onset GBS disease cases continue to occur, leading to acute clinical complications especially for preterm infants [12]. There is a requirement for a rapid diagnostic test for GBS that can be performed in a labour ward setting to ascertain the GBS colonisation status of women in labour, those in preterm labour or women who have not had prenatal care. For these women, culture-screening is not useful because of the time it takes to obtain test results. Several reports demonstrate that real-time PCR is a rapid, more sensitive method than standard culture for determining the intrapartum GBS colonisation status [13-17]. As a result, intrapartum antibiotic prophylaxis can be administered more effectively, thereby reducing the transmission rates of GBS to infants and consequently lowering infant morbidity and mortality rates.

The objective of this study was to develop a rapid nucleic acid diagnostic test for GBS that can be performed in a wide variety of labour and delivery settings. The real-time PCR test developed in this study targets the bacterial *ssrA *gene which was previously shown to be a versatile diagnostic target for important food and clinical pathogens [18,19]. The performance of the *RiboSEQ *GBS test was compared to the commercial FDA-cleared BD GeneOhm™ StrepB Assay (Becton, Dickinson and Company, New Jersey, USA). The results from these tests were correlated with the results of microbiological analyses of the samples.

## Methods

### Collection of specimens

Vaginal swabs from pregnant women (n = 39) were sourced from the Dept. of Obstetrics and Gynaecology, University College Hospital Galway (UCHG), Ireland. Ethics consent was obtained for the study from the Research Ethics Committee at UCHG for vaginal swabs only. Duplicate vaginal swabs were collected into Amies transport medium (Sarstedt, Nümbrecht, Germany), transported to the laboratory at ambient temperature within 3 to 4 hours of collection and processed immediately.

Vaginal swab specimens from pregnant women (n = 120) were purchased from The New England Life Science Group (NELSG) (Los Osos, CA, USA), a clinical services organization. These specimens were remnant swabs screened for GBS colonisation by USA hospital laboratories as part of routine prenatal care. GBS was identified in these swabs at source by genital screen cultures or by selective GBS culture. The remnant specimens were frozen within 1 - 3 days of sampling and shipped on dry ice to our laboratory. A proportion of 90% GBS-positive specimens (n = 107) was requested from NELSG. Those undertaking the performance evaluation of the nucleic acid tests were blinded to the results of the US microbiological analysis while the study was in progress.

### Culture

One of the duplicate antenatal specimens collected from UCHG was used for microbiological evaluation. The swab was inoculated into 7 ml LIM broth (Todd-Hewitt broth with 15 μg/ml nalidixic acid and 10 μg/ml colistin) (LIP Diagnostic Services, Galway, Ireland), incubated overnight and subcultured onto TSA + 5% sheep blood agar (LIP) for 18 - 24 h at 37°C according to CDC recommendations for GBS culture processing. Presumptive GBS colonies showing β-haemolysis were confirmed using the catalase test and antigen detection (Streptococcal Grouping kit, Oxoid, Cambridge, UK). Plates showing no colony growth were reincubated for a further 24 hours and reinspected. Remnant specimens collected from US sites were screened at source for GBS using the CDC-recommended method described above or by genital culture including LIM broth culture.

### Sample preparation

The commercially available crude lysis kit BD GeneOhm™ Lysis Kit (BD, NJ, USA) was used for sample preparation. The second swab of duplicate UCHG specimens and remnant swabs were vortexed for 2 min in 1 ml of sample buffer. Four hundred microlitres of the suspension was transferred into the lysis tube and lysed by mechanical disruption with silica beads according to manufacturer's instructions. Lysates were stored at -20°C until required.

### Construction of IAC

An internal amplification control (IAC) was constructed using the composite primer approach described by Hoorfar et al. [20,21] and O'Grady et al. [19]. An internal amplification control consisting of heterologous DNA cloned into a plasmid vector was included in the *RiboSEQ *GBS test to identify false negative test results caused by PCR inhibition. The IAC is co-amplified with the GBS target and detected by an IAC-specific hybridization probe (Table 1) included in the real-time PCR reaction.

**Table 1:** Oligonucleotide primers and hybridization probes used in this study

Name	Function	Sequence 5' - 3'
gbsU3F	Forward primer	GACAGGCATTATGAGGTA
gbsU4R	Reverse primer	GCTAATATATTTGTCTACAAC
CompF	Forward composite primer for IAC generation	GACAGGCATTATGAGGTAATACCCAACTTGGAATG
CompR	Reverse composite primer for IAC generation	GCTAATATATTTGTCTACAACTCTTCACCAGAATAAAATTG
tmUF	Universal bacterial *ssrA *forward primer	GGGG(A/C)(C/T)TACGG(A/T)TTCGAC
tmUR	Universal bacterial *ssrA *reverse primer	GGGA(A/G)TCGAACC(A/G)(C/G)GTCC
f1GBS-Flu	GBS-specific hybridization probe	TTGCGTTTTGCTAGAAGGTCTTA - Flu
f2GBS-LC640	GBS-specific hybridization probe	LC640- TATCAGCAAACTACGTTTGGCT - Ph
ALS1-Flu	IAC-specific hybridization probe	TGAATGTATCCCCTGGA - Flu
ALS1-LC705	IAC-specific hybridization probe	LC705 - TGGCACTGGTACCATCTAA - Ph

### *RiboSEQ *GBS real-time PCR test

The *ssrA *genes of 10 GBS strains which represent 7 serotypes including the 5 most commonly occurring serotypes (Ia, Ib, II, III, V) were sequenced (Sequiserve, Vaterstetten, Germany) and aligned with other related streptococci (Table 2) *ssrA *sequences generated in this study or available on the tmRNA website [22]. From these alignments, oligonucleotide primers gbsU3F and gbsU4R were designed to amplify a 293 bp PCR product from the GBS *ssrA *gene. A fluorescently labelled hybridization probe pair (f1GBS-Flu and f2GBS-LC640) was designed for the detection of GBS. BLAST (Basic Local Alignment Search Tool) analysis of the hybridization probe sequences was performed to confirm *in silico *specificity of the probes for the detection of GBS.

**Table 2:** Bacterial species and strains used to test the specificity of the *RiboSEQ *real-time PCR test

GBS	Serotype	Collection ref no	Stool Panel	Collection ref no
*Streptococcus agalactiae*	Ia	BCCM 15081	*Escherichia coli*	DSM 30038
*Streptococcus agalactiae*	Ib	BCCM 15082	*Morganella morganii*	DSM 30164
*Streptococcus agalactiae*	Ic	BCCM 15083	*Proteus mirabilis*	DSM 4479
*Streptococcus agalactiae*	II	BCCM 15084	*Klebsiella pneumoniae*	Clinical isolate
*Streptococcus agalactiae*	III	BCCM 15085	*Escherichia hermannii*	DSM 4560
*Streptococcus agalactiae*	IV	BCCM 15086	*Escherichia vulneris*	DSM 4564
*Streptococcus agalactiae*	V	BCCM 15087	*Aeromonas hydrophila*	DSM 30015
*Streptococcus agalactiae*	Ib	BCCM 15090	*Citrobacter freundii*	DSM 30039
*Streptococcus agalactiae*	III	BCCM 15094	*Enterobacter cloacae*	ATCC 13047
*Streptococcus agalactiae*	III	BCCM 15095		
			**Respiratory Panel**	
**Strep spp**			*Staphylococcus aureus*	DSM 11965
*Streptococcus dysgalactiae*		DSM 6176	*Staphylococcus epidermidis*	ATCC 27626
*Streptococcus oralis*		DSM 20627	*Pseudomonas aeruginosa*	ATCC 10145
*Streptococcus pneumoniae*		DSM 11865	*Klebsiella oxytoca*	DSM 5175
*Streptococcus parasanguinis*		DSM 6778		
*Streptococcus intermedius*		DSM 20573	**Neisseria Panel**	
*Streptococcus salivarius*		DSM 20560	*Neisseria meningitidis*	DSM 10036
*Streptococcus uberis*		DSM 20569	*Neisseria lactamica*	DSM 4691
*Streptococcus mitis*		DSM 12643	*Neisseria meningitidis*	ATCC 13077
*Enterococcus faecalis*		DSM 20371	*Neisseria cinerea*	DSM 4630
*Enterococcus faecium*		DSM 20477		
*Streptococcus gordonii*		DSM 6777	**Sepsis Panel**	
*Streptococcus mutans*		DSM 20523	*Streptococcus sanguis*	DSM 20567
*Streptococcus pyogenes*		DSM 20565	*Streptococcus porcinus*	DSM 20725
*Streptococcus anginosus*		DSM 20563	*Serratia marcescens*	DSM 1608
			*Enterobacter aerogenes*	DSM 30053
**Genital Panel**			*Staphylococcus haemolyticus*	DSM 20263
*Acinetobacter lwoffii*		DSM 2403	*Streptococcus bovis*	DSM 20480
*Lactobacillus acidophilus*		DSM 20079		
*Lactobacillus fermentum*		DSM 20055		
*Mobiluncus curtisii*		DSM 2711		
*Mobiluncus mulieris*		DSM 2710		

Real-time PCR was performed in a 20 μl reaction volume on the LightCycler^® ^instrument using the "LightCycler^® ^FastStart DNA Master HybProbe" kit (Roche Diagnostics, Mannheim, Germany). Each reaction contained reagents to final concentrations of: 5 mM MgCl_2_, 0.5 μM each primer (Table 1), 0.2 μM each hybridization probe (Table 1), 0.5 U uracil-DNA glycosylase (Roche). Template was added in 2 μl volumes and IAC was added as 100 recombinant plasmid copies per reaction. Thermal cycling parameters consisted of 95°C denaturation for 10 min followed by 50 amplification cycles of 95°C for 10 s, 50°C for 15 s and 72°C for 10 s. Melting profiles were run between 40°C and 80°C at a transition rate of 0.1°C/s.

### Analytical sensitivity of the *RiboSEQ *GBS real-time PCR test

The limit of detection (LOD) was established using crude lysate extracted from serial dilutions of overnight GBS culture (BCCM 15081) using the IDI Lysis kit (GeneOhm Sciences, Canada). Colony forming units (cfu) per dilution were established by triplicate plate counts and real-time PCR reactions included GBS crude lysate template containing between 10^5 ^and 10^-1 ^cell equivalents.

### BD GeneOhm™ StrepB Assay

The performance of the *RiboSEQ *GBS real-time PCR test for the detection of GBS in clinical samples was benchmarked against the performance of the commercially available FDA-approved BD GeneOhm™ StrepB Assay which targets the CAMP factor B (*cfb*) gene. The assay was performed on the SmartCycler^® ^instrument (Cepheid, California, USA) according to manufacturer's instructions. Briefly, 25 μl of diluent was added to each Master Mix reaction tube. Positive and negative control tubes were included in each run. Sample crude lysates were added to the Master Mix tubes in 1.5 μl volumes, the tubes were briefly centrifuged and placed in the SmartCycler^® ^instrument. Assay runs were performed using the BD GeneOhm™ StrepB Assay-specific software. The BD GeneOhm™ StrepB Assay takes approximately 60 min to run including sample preparation.

## Results

### Analytical sensitivity and specificity of the *RiboSEQ *GBS real-time PCR test

The limit of detection of the *RiboSEQ *GBS test was determined by three independent experimental assessments and consistently detected in the range of 1 - 10 cell equivalents per reaction. The inclusion of an internal amplification control at a concentration of 100 plasmid copies per test did not lower the LOD (data not shown). At high GBS template concentrations (≥ 10^5 ^cell equivalents/reaction) the amplification of internal control target was occasionally inhibited due to competition between GBS target DNA and IAC in the PCR reaction.

Specificity studies were performed using DNA prepared from a panel of 10 GBS strains representing 7 serotypes including 5 common serotypes and 42 related streptococci and other species found in the genital tract environment (Table 2). All 10 GBS strains were detected. The test was negative for all non-GBS species listed in Table 2. The DNA extracted from non-GBS species was confirmed to be amenable to real-time PCR with universal primers tmUF and tmUR (Table 1) which were designed to amplify the bacterial *ssrA *gene.

### Evaluation of the *RiboSEQ *GBS test in clinical samples

Performance of the *RiboSEQ *GBS test was compared to the results of microbiological culture methods for the isolation of GBS. Test results were also compared to those obtained using the commercial BD GeneOhm™ StrepB Assay. Microbiological culture results were used as the gold standard.

A total of 159 specimens (39 from UCHG, 120 from NELSG) were tested by microbiological culture, *RiboSEQ *GBS real-time PCR and BD GeneOhm™ StrepB Assay. Microbiological culture identified 111 samples as GBS-positive and 48 samples as GBS-negative.

The prevalence of GBS colonisation among the specimens collected from UCHG was 10.3% (4/39) using the GBS selective culture method. Calculation of prevalence of GBS colonisation in the NELSG samples is not applicable since this population of specimens was selected to be predominantly GBS-positive and not representative of the true prevalence among pregnant women.

In 146 samples, the results of all three tests showed 100% correlation, where 104 samples were identified as GBS-positive and 42 samples were identified as GBS-negative. In 95% of samples the two nucleic acid diagnostic test (NAD) results agreed (151/159).

Discrepant results were obtained in a total of 13 samples after retesting the crude lysate. In 5 samples, NAD test results agreed but conflicted with the microbiology results. Microbiological culture identified 2 samples as GBS-negative which the NAD tests identified as GBS-positive. Three samples were determined as GBS-positive by culture and tested negative in the NAD tests.

In one sample, the *RiboSEQ *GBS test was negative for a sample that was GBS-positive by both culture and BD GeneOhm™ StrepB Assay. PCR inhibition was not apparent since the IAC gave a positive signal.

For 6 samples, the BD GeneOhm™ StrepB Assay results disagreed with concurring culture and GBS real-time PCR results (3 false positives, 3 false negatives).

In another sample, both the IAC and the *RiboSEQ *GBS test results were negative, indicating PCR inhibition. This specimen was identified as GBS-negative by both culture and BD GeneOhm™ StrepB Assay with no indication of PCR inhibition. However, the discrepancy was resolved for this sample by increasing the IAC to 200 plasmid copies in the *RiboSEQ *GBS real-time PCR reaction which yielded a positive result for the IAC for this sample.

Table 3 shows the sensitivity, specificity, predictive values and likelihood ratios for the *RiboSEQ *GBS test and the BD GeneOhm™ StrepB Assay using microbiological culture for the identification of GBS as the gold standard. Confidence intervals (CI) are stated at the 95% level.

**Table 3:** Sensitivity, specificity, predictive values (PPV, NPV) and likelihood ratios +LR, -LR) for the *RiboSEQ *GBS test and the BD GeneOhm™ StrepB Assay

		*RiboSEQ *GBS test		BD GeneOhm™ StrepB Assay	
		Positive	Negative	Positive	Negative
**Culture^a^**	**Positive**	107	4	105	6
	**Negative**	2	46	5	43
					
	**Sensitivity**	96.4%		94.6%	
	**95% CI**	(90.5 - 98.8%)		(88.1 - 97.8%)	
	**Specificity**	95.8%		89.6%	
	**95% CI**	(84.6 - 99.3%)		(76.6 - 96.1%)	
	**PPV^b^**	98.2%		95.5%	
	**95% CI**	(92.9 - 99.7%)		(89.2 - 98.3%)	
	**NPV^b^**	92%		87.8%	
	**95% CI**	(79.9 - 97.4%)		(74.5 - 94.9%)	
	**+LR^b^**	23.1		9.1	
	**95% CI**	(5.9 - 89.9)		(3.9 - 20.8)	
	**-LR^b^**	0.04		0.06	
	**95% CI**	(0.01 - 0.1)		(0.03 - 0.13)	

Calculated using programme available at http://faculty.vassar.edu/lowry/clin1.html

^a ^using culture as the gold standard (n = 159)

^b ^sample population comprised of random (n = 39) and non-random (n = 120) specimens

## Discussion

In a systematic review of the accuracy and rapidity of various intrapartum GBS colonisation tests, real-time PCR was identified as one of two feasible methods for maternal intrapartum screening [23]. Several studies have demonstrated the speed, sensitivity and accuracy of real-time PCR tests for the detection of GBS in pregnant women [14,24,25]. Recently, a real-time PCR assay for the detection of ST-17 serotype III, a virulent strain of GBS associated with neonatal invasive infections was developed [26]. Clinical studies have shown an increase in sensitivity for real-time PCR tests in comparison to the gold standard culture method [13,27]. Higher detection rates of real-time PCR tests may be explained by the inability of culture to detect low numbers of organisms, the presence of antagonistic organisms, or possibly the detection of non-viable cells [10].

A relatively low carriage rate obtained by culture for GBS in this study was similar to the carriage rate of 8% reported in a recent study where vaginal swabs from pregnant women were screened for GBS [28]. Ideally, both vaginal and rectal swabs are required to ensure the highest possible GBS detection rate [29]. However, for this study rectal samples were not available since the specimens were collected during routine prenatal care for the purpose of general microbiological screening. Additionally, only colonies showing β-haemolysis were tested with the catalase and latex agglutination tests. The CDC method also recommends testing characteristic colonies without β-haemolysis.

In this study we developed a qualitative real-time PCR test for the detection of GBS that is rapid (75 min including sample preparation) and capable of detecting 1 - 10 genome copies of GBS. The sensitivity and specificity achieved with the test in comparison to culture were 96.4% (CI 95% 90.5 - 98.8) and 95.8% (CI 95% 84.6 - 99.3), respectively. These values compare well with previously published sensitivities and specificities of real-time PCR assays for GBS [14,15,29,24,27] (Table 4). In our hands, the *RiboSEQ *GBS real-time PCR test performed slightly better than the commercial FDA-approved BD GeneOhm™ StrepB Assay (sensitivity 94.6% (CI 95% 88.1 - 97.8), specificity 89.6% (CI 95% 76.6 - 96.1)). Two previous studies evaluated the clinical performance of the BD GeneOhm™ StrepB Assay and reported a sensitivity of 94% and a specificity of 95.9% for direct detection [14] and a sensitivity of 92.5% and a specificity of 92.5% after 4 h selective enrichment [16].

**Table 4:** Comparison of sensitivities and specificities of a selection of diagnostic real-time PCR assays for GBS (using culture for GBS as gold standard)

Reference	Assay name	Target gene	Sensitivity % (95% CI)	Specificity % (95% CI)
This study	*RiboSEQ *GBS	*ssrA*	96.4 (90.5 - 98.8)	95.8 (84.6 - 99.3)
Bergeron et al (2000)	Not specified	*cfb*	97 (82.5 - 99.8)	100 (94.2 - 100)
Bergseng et al (2007)	Not specified	*sip*	97 (90 - 99)	99 (97 - 100)
Davies et al (2004)	IDI-Strep B	*cfb*	94 (90.1 - 97.8)	95.9 (94.3 - 97.4)
Gavino and Wang (2007)	GeneXpert GBS	*cfb*	95.8 (76.9 - 99.8)	64.5 (45.4 - 80.2)
Uhl et al (2005)	LightCycler Strep B	*ptsI*	100 (88.8 - 100)	96.9 (92.2 - 99.1)

Test results from the real-time PCR tests correlated in 95% of samples. In five samples the culture results disagreed with concurring NAD test results. Two of these 5 samples were identified as culture-negative but GBS-positive by both real-time PCR tests. Increased sensitivities for real-time PCR diagnostic tests over the standard culture method have been reported in other studies [8,13,14] and can be a result of detection of non-viable cells, low bacterial burden [13,16] or the presence of antagonistic microorganisms which inhibit growth in culture [30,31]. In three cases, the PCR tests were negative for culture-positive samples. However, this discrepancy may be due to misidentification of GBS during culture screening. Two of these specimens were screened by genital culture and GBS growth may have been misidentified. The third sample was a UCHG duplicate swab in which the DNA may have been degraded. This sample had been stored at 4°C for 5 days before processing for real-time PCR.

Due to the dynamic status of vaginal GBS colonisation, screening intrapartum is the most accurate method of predicting the GBS colonisation status [2,32]. Screening for GBS at the time of delivery allows antibiotics to be used more effectively thereby rationalising their use and minimising associated risks. The value of a rapid and accurate test for GBS colonisation is especially apparent in cases of premature delivery of <35 weeks gestation when screening results may not be available and babies are at greatest risk of developing early-onset GBS disease.

In spite of the advantages of sensitivity, specificity and fast turnaround time, the complexity of real-time PCR technology poses limitations to its widespread application in clinical laboratory testing. Generally, it requires trained personnel and dedicated laboratory areas. In addition, specimens are often batched for analysis prolonging turnaround time. However, these limitations have been reduced through the development of fully integrated and automated *in vitro *diagnostic platforms which are moderately complex in their operation and can be performed in an on-demand schedule [33]. These platforms provide the potential for diagnostic tests such as the *RiboSEQ *GBS test to be employed in hospital laboratories and near-patient settings where a rapid and reliable diagnosis is most valuable.

## Conclusion

In summary, a rapid and sensitive qualitative test for the detection of GBS in vaginal swabs by real-time PCR was developed. The test targets the bacterial *ssrA *gene which was previously shown to be a suitable and versatile diagnostic target for important food and clinical pathogens. The method has potential to be employed as a screening and/or diagnostic test for use in clinical laboratories where an assessment of the patient's GBS colonisation status at the time of delivery is required without delay.

## Competing interests

Some of the authors are inventors of a platform technology based on the *ssrA *gene (*RiboSEQ*) for bacterial identification that is currently going through PCT patent filing.

## Authors' contributions

MW: Performed the experimental work described in the manuscript and contributed intellectually to the design of the real-time PCR *RiboSEQ *GBS test. Lead author in preparing the manuscript. CM: Performed the experimental work described in the manuscript and contributed to manuscript preparation. VS: Contributed to clinical study design, collection of specimens for the study and analysis and interpretation of results. JM: Contributed to clinical study design, collection of specimens for the study and critical analysis and interpretation of results. TB: Member of the project management team responsible for the study concept and design, critical review of the experimental work and review of the manuscript. MM: Member of the project management team responsible for the study concept and design, critical review of the experimental work and preparation and critical review of the manuscript. TS: Member of the project management team responsible for the study concept and design, critical review of the experimental work and preparation and critical review of the manuscript.

## Pre-publication history

The pre-publication history for this paper can be accessed here:

http://www.biomedcentral.com/1471-2334/9/148/prepub
